# Towards Understanding Contemporary Gambling Advertising in Sub‐Saharan Africa

**DOI:** 10.1002/puh2.70017

**Published:** 2024-12-09

**Authors:** Emmanuel Badu

**Affiliations:** ^1^ Collaboration for Evidence, Research and Impact in Public Health, School of Population Health Curtin University Perth Western Australia Australia

**Keywords:** betting, commercial determinants, gambling advertising, gambling, sub‐Saharan Africa

## Abstract

Gambling advertising has become ubiquitous in sub‐Saharan Africa influencing social norms and attitudes towards gambling. The delay or failure of governments to effectively regulate gambling advertising poses a significant threat to public health. This article provides contemporary insights into gambling advertising, its forms, mechanics and influence on gambling behaviour. It provides contextual understanding to policy‐makers, health advocates and public health actors, including a call to action for effective regulation. With sub‐Saharan Africa becoming important to the gambling industry for market growth and profits, there is the need for public health actors to instigate advocacy for strong regulations and enforcement of gambling advertising.

## 1 Background

Commercial gambling industry is driving gambling behaviour and gambling‐related harm in sub‐Saharan Africa (SSA) [1, 2]. The liberalisation of gambling markets and the subsequent establishment of gambling regulations [3] provided the legitimacy for the gambling industry to rapidly expand their markets through innovation such as the evolution of diverse modes of gambling products [4]. Spurred on by the weak and outdated gambling advertising regulations [3], gambling operators have aggressively been promoting gambling as a legal, social and culturally acceptable leisure activity and risk‐free way to make quick money. In the last decade, gambling advertising and gambling opportunities have become a common feature in countries in SSA [4]. Much of this advertising is particularly linked to sports betting which is the commonest form of gambling [4, 5, 6, 7, 8, 9]. The delay and failure to effectively regulate gambling advertising has contributed to gambling advertisements and gambling opportunities becoming widely available in everyday spaces and on all forms of media.

In this article, I provide a contemporary public health insight into gambling advertising from the international literature, including a contextual understanding of how gambling operators through pervasive advertising are driving gambling behaviour in SSA. I conclude with a call to action by public health researchers, advocates and policy‐makers.

## 2 Main Themes and Narratives Used in Gambling Advertising

Advertisements are a strong normalising influence on attitudes, behavioural intentions and ultimately the execution of the expected behaviour of their target audience [10]. They are the key mode by which the gambling industry constructs social norms, influences perceptions and public discourses, including what meanings the public attach to products. In the case of gambling products, industries employ themes, metaphors and images to rework cultural and societal meanings and appeal about these products [11]. Repeated exposure to gambling messages and gambling opportunities would likely increase societal acceptance and legitimacy of gambling as a legal pursuit of pleasure, excitement and leisure [12, 13] and has been reported to create a sense of fear of missing out (FOMO) among bettors in Kenya [14].

The international evidence suggests that although gambling operators may create advertisements for specific jurisdictions, the underlying themes and narratives employed in the advertising content to influence gambling behaviour are similar [15]. A useful theory that helps understand the common themes and narratives used in gambling advertising is the symbolic consumption theory, which asserts that the consumption of goods depends more on their social meaning to consumers than their functional utility [16]. Symbolic consumption is premised on products as causes of behaviour rather than responses to behaviour, and advertising is to create symbolic effects that emphasise social appeal and elicit consumption and use of products [16]. In turn, people use symbols associated with advertising and gambling products to assign social identities and meaning for their behaviour, reinforcing that gambling products are key determinants of behaviour [11, 16]. Thus, in an environment where gambling advertising is widespread and poorly regulated and gambling opportunities abound [10], people are more likely to engage in gambling relying on the symbolic cues as portrayed in gambling advertisements to guide their behaviour and outcome expectations [17].

Advertising contents are positively framed and embed themes that glamorise gambling as fun, desirable, social, legal, risk‐free and trustworthy activity [15]. Gambling brand advertisements employ imageries of financial success and gender stereotypes which appeal to young males [11]. These include imageries of winning more money to afford a glamorous lifestyle, becoming more attractive, having options to enjoy with women and gaining more power over life including people's choice of luxury activities. They also incorporate thrills and excitement themes such as winning lots of cash after placing a bet which appeals to young males [11]. For instance, the gambling industry in sub‐Saharan Africa has been reported to use media to cover stories of customers who win substantial amounts in a bid to create more visibility and entice more young people to gamble [18].

In addition, gambling operators imbue advertisements with sports culture and practices such as bonding with friends, loyalty and invoke emotionally charged imagery, including fans celebrating a goal or a win, cheering, or waving team flags to draw in consumers to bet [19]. For example, a study in Malawi found that gambling advertisements used themes that appealed to hypermasculinity such as sports knowledge, skills, competition and triumph over others to appeal to young males [18]. By embedding sports rituals and practices such as friendships and togetherness, operators invoke symbolic consumption ideals seeking to deepen and exploit the emotional relationship or connection between the bettor and the team they support [11]. This links to the idea that winning could bring friends and acquaintances closer together and builds on concepts of fraternity or “brotherhood” in sports.

Moreover, sports betting operators present betting as a rational calculated game in which people who are smart and able to study and predict games can win big rewards [14, 15, 19]. Betting advertisements overemphasise the illusory sense of control, portraying bettors as people having control over the outcome of the game and the chances of winning money while minimising any risks of losses [20]. Gambling operators will often use celebrities to reinforce consumers’ loyalty to sports teams [14, 15], minimise risks of gambling and enhance the credibility and trustworthiness of the gambling brand [20]. The incorporation of such persuasive themes related to fun, big legal money, control and knowledge of the game encourages and sustains the behaviour while enhancing market growth and profits for the industry.

The available literature also points to the use of humour and comedy in adverts as attention‐grabbing and risk‐lowering strategy normalising gambling as an exciting risk‐free activity [15]. The industry, fully aware that young people, who are mostly their target audience, will tune out or skip a straightforward advertisement, would begin their advertisements with and infuse humour throughout the advertisement to draw and sustain the attention of their target audience. The use of humour minimises any risks or the susceptibility to negative consequences associated with gambling behaviour, while overwhelmingly highlighting the excitement and benefits to be derived from gambling [11]. Using humour could further be likened to a bait for social media networks, where young people do share such funny videos and memes peer‐endorsing such products and normalising gambling behaviour.

## 3 Forms of Gambling Advertising and Where They Occur

Gambling advertisements come in various forms, including on television, radio, sporting events and on online and digital platforms. It goes beyond commercial breaks during live broadcast to include display of logos on sports team shirts, commentator live mentioning, pitch side digital displays, direct messages about in‐game odds and social media posts during games [15]. The international literature on gambling advertising shows that gambling operators have evolved in the delivery and placement of advertisements, shifting from traditional forms of advertising on television and radio to extensive and complex advertising on the Internet, including via social media platforms [10, 15, 19, 21]. For instance, a recent systematic review of the evidence noted that the volume and spend on advertising by sports betting operators was increasing in the face of restrictions to advertising on television in the United Kingdom during games, strong indication that the gambling industry retains a high level of presence and exposure through other forms of advertising during and off games [15].

Although some form of restrictions of gambling advertising on television may be in place and may vary by country, digital online advertising is often poorly regulated [22]. One such area of concern is the normalisation of betting and its intersection with sports consumption on online platforms [23]. Gambling advertising is prominent on online sports media which tend to be affiliates of betting operators. Affiliate advertising can include placement of betting banners on online sports media and sports streaming channels which can lead bettors to bet on the betting operators’ website [23]. These sports platforms, many of whom are illegal and poorly regulated in SSA region, serve as channel for advertising circumventing many of the existing advertising restrictions.

Social media posts by gambling operators commonly highlight news, events and wins, aimed at building brand awareness and are intended to nudge bettors to place bets impulsively during games [19]. Digital online and social media platforms are used to disseminate inducements and financial incentives such as offers, free bets, bonuses and promotional deals to nudge consumers to place bets [14, 15]. Operators employ direct advertising messages with high frequency of use during games. These personalised advertising may include emailing, texting or social media posts of customised bets with link to the operators’ websites, sports‐related content, including expert commentary or comments from sportspeople [15, 19]. The enhanced user experience and interactivity on online platforms play a crucial role in sustaining consumers engagement on these platforms. Marketing algorithms and techniques on social media platforms allow gambling operators to personalise adverts and instil strong emotive appeal towards products and brands [19]. These enable consumers to like, comment, recommend and share gambling industry posts across their own online community, normalising gambling and sometimes exposing under‐age populations to gambling in the process.

## 4 The Features and Mechanics of Gambling Advertising

Gambling operators employ in‐play advertising, also known as real‐time gambling or live action gambling, to encourage repeat betting during a game [19]. In‐play gambling is promoted by operators as a thrilling opportunity for bettors to win or better their chances of winning in real‐time during the game. Operators entice gamblers with enhanced odds that are updated and promoted during games, often with higher than the usual returns, to attract and sustain repeat betting behaviour [24]. These complex odds have higher profit margins for operators than the simple gambles, yet are promoted by operators as a better and easier option to enhance punter's chances of winning [25]. Bettors receive reminders about in‐play odds during games with links to expert analysis, detailed predictions, historical game information, including upcoming events and information on how to bet. These are aimed at influencing consumers sense of control over the game and ultimately nudging them to place more bets [20]. As shown in a recent study, odds advertising does positively influence gambling intention and attitudes especially among young males compared to betting adverts that do not include odds [26].

Gamblers are offered multiple options to place bets and/or adapt bets, whereas the game is in session as opposed to fixed‐odds gambling where bettors place a wager on the outcomes of a game before the game starts [24]. For example, gamblers can bet in‐play on half time score, number of yellow cards, first or next goal scorer, a specific player scoring, a specific player scoring and a team winning, and on the final match scoreline [24, 25]. In‐play advertisement uses narratives that would appear more urgent than necessary including reminding consumers they can boost their chances of winning additional money using all the expert advice on the game playing, their own knowledge of the game and the enhanced odds that are available within the specified time period [24, 25]. Such limited‐time offers have been reported to create FOMO and a sense of urgency to bet [14]. The very nature of in‐play gambling, the urgency and ubiquity of advertising associated with this type of gambling, reinforces and facilitates continuous, impulsive and excessive gambling behaviour maximising profits for gambling operators [24].

## 5 Implications for Public Health Action

This article provides a synthesis of the contemporary areas in gambling advertising. It raises awareness about the sophisticated approaches and techniques employed by gambling operators to create and sustain demand for their products. The rapid innovations in online and digital advertising through a borderless internet pose the greatest challenge to countries, especially those in SSA. Social media advertising, including paid advertising, organic advertising or peer‐endorsed sharing of memes and funny videos with gambling content, is contributing to positive attitudes towards gambling and the normalisation of gambling behaviour. The social concern for gambling is rising as the consequences of gambling become apparent in people's lives, their communities and societies. What is missing, however, is a dedicated public health response riding on these societal sentiments to address the growing challenge of gambling in the region. Strong sustained advocacy to counter gambling industry influence is urgently required.

Recent gambling advertising restrictions introduced in Belgium [27] that explicitly focus on protecting public health and society from the dangers of gambling advertising offer useful practical guide in what actions could be implemented, and why government action should be prioritised. In 2023, Belgium prohibited traditional and online gambling advertising, including on‐demand streaming on the internet, personalised advertisement via email, SMS or social media [27]. Belgium's prohibitions have led to gambling advertising disappearing from public spaces. However, recent evidence suggests that gambling operators in Belgium are adopting novel tactics to circumvent and exploit loopholes in the restrictions, including investing in internet search engine advertising and increasing their corporate social responsibility communication activities for more media coverage [28]. These observations highlight the sophistication and predatory nature of the gambling industry in driving consumption and profits. Accordingly, a strengthened regulatory framework and enforcement mechanism are urgently needed to reverse the rapid normalisation of gambling in the region.

Lastly, it is imperative to refocus gambling research in the region from individual psychological factors to robust research demonstrating how the gambling industry and its affiliates and powerful vested interests are driving gambling behaviour and harms to inform appropriate regulatory action.

## Author Contributions

**Emmanuel Badu**: conceptualisation, writing–original draft, writing–review and editing, investigation.

## Conflicts of Interest

The author declares no conflicts of interest.

## Data Availability

Data sharing is not applicable to this article as no new data were created or analysed in this study.
